# In Vivo Assessment of the Photoprotective Potential of Underutilized Carob Fractions by Using *Caenorhabditis elegans*

**DOI:** 10.3390/plants14213257

**Published:** 2025-10-24

**Authors:** Héctor Gómez-Llorente, Samuel Furones, Noelia Castillejo, Sara Tortajada, Samuel Verdú, Raúl Grau, Édgar Pérez-Esteve, José M. Barat

**Affiliations:** Instituto Universitario de Ingeniería de Alimentos-Food UPV, Universitat Politècnica de València, Camino de Vera s/n, 46022 Valencia, Spain; hecgollo@upv.es (H.G.-L.); sfurher@etseamn.upv.es (S.F.); noelia.castillejo@upct.es (N.C.); satorma1@alumni.upv.es (S.T.); saveram@upvnet.upv.es (S.V.); rgraume@tal.upv.es (R.G.); edpees@upv.es (É.P.-E.)

**Keywords:** *Ceratonia siliqua* L., antioxidant activity, solar protection factor, erythema transmission, lethality

## Abstract

The revalorization of agro-industrial by-products is a key strategy for promoting sustainability and the circular economy. This study assessed the photoprotective potential of underutilized carob (*Ceratonia siliqua* L.) fractions, including unripe and mature pods, leaves, and seed tissues, through chemical characterization, in vitro assays, and in vivo validation. Extracts showed high polyphenol contents (up to 4.8 g GAE/100 g) and strong antioxidant activity (up to 45 g TE/100 g). Photoprotective properties were confirmed by a solar protection factor of up to 17 and erythema transmission values of 3–6, indicating efficient UV absorption and anti-inflammatory potential, which together support overall skin protection. To validate these effects under physiological conditions, *Caenorhabditis elegans* was used as an in vivo model under three exposure modalities: complete exposure (contact and ingestion), the barrier effect (UV shielding by the medium), and the physiological effect (systemic protection after ingestion). Seed episperm and unripe pods showed the highest efficacy. Notably, complete exposure reduced UV-induced lethality from 98% to below 50%, mainly due to the barrier effect. This is the first report demonstrating the photoprotective activity of carob by-products in *C. elegans*, supporting their potential as natural ingredients for cosmetic and nutraceutical applications, and contributing to the sustainable revalorization of local agricultural residues.

## 1. Introduction

Global agricultural waste remains a critical challenge, with approximately 1.05 billion tons discarded in 2024 and projections indicating a rise to 2.6 billion tons by 2030 [1,2]. This waste of resources not only represents missed opportunities for value creation but also reflects inefficient utilization and contributes substantially to environmental impact [3]. In response to these challenges, the United Nations’ 2030 Agenda and its Sustainable Development Goals (SDGs) identify sustainability, efficient resource use, and circular economy practices as key pillars for guiding future global development [4]. Within this framework, the agro-industrial sector is increasingly adopting strategies focused on the valorization of by- and underutilized products and the implementation of zero-waste models. Achieving these goals requires research aimed at characterizing the composition, bioactive properties and functional potential of agro-industrial residues. By exploring novel applications, these underutilized materials can be transformed into valuable ingredients, expanding their range of uses and fostering innovation across agricultural and industrial systems, while also contributing to the stabilization of supply chains and market demand, and offering farmers more reliable and attractive incentives for sustainable production [5,6].

Native to the Mediterranean, carob is highly resilient to drought, heat, and pests, making it well-adapted to the challenges posed by climate change and suitable for cultivation in arid and semi-arid regions [7]. Despite its agronomic and ecological advantages, the crop remains largely underutilized: while the seed endosperm is industrially processed into locust bean gum, most of the fruit (around 95%), including the episperm and germ of seeds, pods, and leaves, is often discarded [8].

One of the main challenges for human health is the continuous exposure to solar ultraviolet (UV) radiation during daily outdoor activities [9]. This persistent exposure generates reactive oxygen species, leading to oxidative stress, DNA damage, premature skin aging, and an increased risk of skin cancer [10]. While synthetic UV filters in sunscreens are commonly used during intentional sun exposure, such as sunbathing, daily use body creams may also contribute to mitigating the effects of continuous UV exposure [11].

In all the products, incorporating botanical extracts has been shown to be a complementary strategy for enhancing their functional effects [12]. In this regard, in vitro analysis as total polyphenol content (TPC), antioxidant activity, solar protection factor (SPF) and erythema transmission of the extracts are routinely evaluated before adding to the cream as the ability of plant polyphenols to mitigate oxidative stress and enhance cellular defense mechanisms against UV exposure are known [13,14,15]. These functional properties would be particularly interesting in carob as their fruit or leaves have been identified as rich sources of bioactive compounds, such as polyphenols and inositols, with promising photoprotective activity [16,17,18].

Despite the high bioactivity reported in vitro, actual efficacy depends on factors such as bioavailability and metabolism [19], making in vivo validation essential. *Caenorhabditis elegans* (*C. elegans*) is a robust model for oxidative stress and UV-induced damage due to its short life cycle, genetic similarity to humans (60–80% gene homology), and suitability for rapid, ethical screening of bioactive compounds [20,21]. This nematode has been successfully employed to evaluate the protective effects of polyphenols such as resveratrol and quercetin, as well as botanical extracts, against UV exposure [22,23,24,25,26]. However, to date, no studies have explored the photoprotective potential of carob fractions in this in vivo model, highlighting a novel application for this underutilized raw material.

Under these considerations, a comprehensive multi-tiered evaluation was carried out, integrating chemical, functional, and biological analyses. First, in vitro assays were used to quantify antioxidant activity and the phenolic composition of the extracts. Photoprotective properties were then assessed through sun protection factor and erythema transmission measurements. Subsequently, an in vivo evaluation using *C. elegans* was conducted to examine survival and physiological responses under UV-induced oxidative stress through three exposure modalities: complete, barrier and physiological. This strategy not only identified the most promising fractions for natural photoprotection but also demonstrated the potential of plant-derived residues to be sustainably revalorized into clean-label solutions, aligned with circular economy principles and the Sustainable Development Goals (SDGs).

## 2. Materials and Methods

### 2.1. Carob Fractions and Reagents

Seed episperm and seed germ were supplied by G.A. Torres S.L. (Valencia, Spain). Carob leaves and whole pods were manually collected from local trees in Enguera (Valencia, Spain), with unripe pods harvested in April and mature pods and leaves collected in August. All carob fractions were vacuum-packed and stored at −18 °C until use. These materials constituted the carob fractions evaluated throughout the study.

Reagents DPPH, Folin–Ciocalteu, fluorescein (FL), trolox, gallic acid, and 2,2′-Azobis(2-methylpropionamidine)dihydrochloride (AAPH) used in the assays for total phenolic compounds and antioxidant activity were purchased from Sigma-Aldrich (Madrid, Spain). Sodium hydroxide (NaOH), sodium hypochlorite (NaClO), H_2_O_2_, and the reagents used for nematode growth (NGM) (NaCl, peptone, agar, and phosphate buffer) were purchased from Sigma-Aldrich (Spain), potassium phosphate buffer, CaCl_2_-2H_2_O, MgSO_4_-7H_2_O, and cholesterol in ethanol), K-medium (KCl and NaCl), and M9 buffer (KH_2_PO_4_, Na_2_HPO_4_, NaCl, and MgSO_4_) were purchased from Scharlab (Barcelona, Spain), except for cholesterol (95% *w*/*w*), which was supplied by Acros Organics (Geel, Belgium).

### 2.2. Preparation of Extracts

To evaluate the functional properties of the carob fractions, a hydroalcoholic extract was prepared following the methodology described by Valverde et al. [27], with minor changes. Briefly, 2 g of sample were homogenized in 15 mL of an ethanol/water solution (50:50, *v*/*v*), corresponding to a solvent-to-sample ratio of 7.5:1 (mL/g), and was subjected to ultrasonic treatment (Elmasonic S 40 H, Singen, Germany) for 10 min at 37 KHz and room temperature (RT) to enhance analyte diffusion into the solvent. After centrifugation at 9000× *g* at RT, the supernatant was collected. This extraction step was repeated twice on the remaining pellet, with the third cycle using an acetone/water mixture (70:30, *v*/*v*). Finally, the three supernatants were mixed, and the organic fraction (ethanol and acetone) evaporated to prevent any nematode toxicity or interference. Finally, the concentrated extracts were resuspended in 25 mL of distilled water, yielding approximately 20% (*w*/*w*) of dry extract relative to the initial sample. The entire crude extract was used for subsequent analyses.

### 2.3. In Vitro Functional Analysis

#### 2.3.1. Total Phenolic Content

The quantification of TPC was performed using the Folin–Ciocalteu method as described by Valverde et al. [27]. An aliquot of 15.8 μL of the diluted extracts was mixed with 158 μL of Folin–Ciocalteu, previously diluted in double-distilled water (1:10, *v*/*v*), in a 96-well microplate. After 3 min of incubation in darkness, 126 μL of sodium carbonate solution at a concentration of 1 M was added, and the mixture was incubated for 1 h in darkness. The microplate was then measured at 765 nm using a VANTAstar microplate reader (BMG LabTech, Ortenberg, Germany). Absorbance values were interpolated from a standard calibration curve prepared with gallic acid in the extraction solution. Results were expressed as grams of gallic acid equivalents per 100 g of sample (g Gallic acid E/100 g). Analysis was conducted three times in triplicate (*n* = 9).

#### 2.3.2. Antioxidant Activity

Antioxidant activity was determined by both the DPPH and ORAC methods using a VANTAstar microplate reader (BMG LabTech, Ortenberg, Germany). In the case of DPPH, 30 μL of the diluted extracts was mixed with 270 μL of DPPH in a 96-well microplate, and the mixture was incubated for 1 h in darkness. The absorbance of the sample was then determined at 515 nm. On the other hand, the ORAC assay using FL was performed as described by Ribes et al. [28] with slight modifications. A volume of 20 μL of diluted sample in 75 mM phosphate buffer (pH 7.4) and 70 μL of 200 nM FL in same buffer was mixed and incubated for 15 min at 37 °C in darkness. Afterwards, 110 μL of 250 mM AAPH diluted in the buffer was added and fluorescence was measured every minute for 60 min, using 485 nm for excitation and 538 nm for emission wavelengths.

For the two methods, values were interpolated from a standard calibration curve prepared with trolox in the extraction solution. Results were expressed as grams of Trolox equivalents per 100 g of sample (g Trolox E/100 g). Analysis was conducted three times in triplicate (*n* = 9).

#### 2.3.3. Solar Protection Factor

The SPF of the carob extracts was determined following Caballero-Gallardo et al. [13] with minor modifications. Extracts were diluted in distilled water (1% and 5% *v*/*v*) and dispensed into 96-well microplates (200 µL per well; optical path of ca. 0.6 cm). Absorbance values were corrected by subtracting the baseline absorbance of the solvent (distilled water) and recorded from 290 to 320 nm (UVB range) using a microplate reader. Finally, SPF was then calculated using Mansur Equation (1) [29].

(1) $$SPF=CF\times \sum_{290nm}^{320nm}EE\left(\lambda \right)\times I\left(\lambda \right)\times Abs(\lambda )$$

In the Mansur equation, CF (correction factor) is an empirical constant set to 10 for standardization [30], EE(λ) × I(λ) are tabulated values from Caballero-Gallardo et al. [13] representing the relative erythemal effectiveness and solar intensity at each wavelength, and Abs(λ) is the spectrophotometrically measured absorbance of the sample at wavelength λ, corrected by subtracting the baseline absorbance of the solvent. All analyses were performed in triplicate (*n* = 3).

#### 2.3.4. Erythema Transmission

Erythema transmission (%) of extracts at concentrations of 1% and 5% in distilled water was assessed from absorbance measurements taken across the 292–372 nm wavelength range using 96-well microplates (200 µL per well; effective optical path of ca. 0.6 cm). Absorbance values were corrected by subtracting the baseline absorbance of the solvent before calculations. Photoprotection indices were subsequently calculated from the transmission (T), which was determined according to Equation (2). Finally, erythema transmission was performed following Equation (3).

(2) $$A=-\log_{10}\left(T\right)$$

(3) $$Transimission\;of\;erythema\left(\%\right)=\frac{Ee}{\sum Fe}=\sum \frac{\left(T\times Fe\right)}{\sum Fe}$$

In Equation (3), Fe represents the erythemal flux, which maintains constant values for wavelengths between 292 and 338 nm. In this study, values were retrieved from Caballero-Gallardo et al. [13]. All analyses were performed in triplicate (*n* = 3).

### 2.4. In Vivo Photoprotective Analysis Using C. elegans

#### 2.4.1. *C. elegans* Strain and Maintenance

The strain used in this study was the wild-type *C. elegans* Bristol strain N2 (Caenorhabditis Genetics Center (University of Minnesota, Minneapolis, MN, USA). Nematodes were cultured on NGM agar plates which were seeded with *Escherichia coli* OP50 at 20 °C, as the nematode food source. Regular subculturing was performed to prevent overcrowding and maintain the population. For all experiments, adult hermaphrodites were synchronized to the same larval stage using an alkaline hypochlorite solution (0.6 M NaOH, 1% NaClO) and then cultured at 20 °C in darkness for 3 d until reaching the adult stage [31], following the method described by Verdu et al. [32].

#### 2.4.2. Effect of Carob Extracts on Lethality Rate of *C. elegans* After UV Exposure

After reaching the adult stage, *C. elegans* populations were used to evaluate the photoprotective effect of different carob fractions (seed germ, seed episperm, mature pods, unripe pods, and leaves). To distinguish between external photoprotection, such as UV absorption by compounds in the surrounding medium, and internal protection mediated by the ingestion and metabolism of bioactive compounds, three exposure conditions were tested. In complete exposure (i), nematodes were incubated with the extracts for 24 h at 20 °C before light irradiation, allowing both the barrier effect of the bioactives in the solution and their ingestion through the natural feeding behavior of *C. elegans*, thus assessing the combined external and internal effects. To evaluate the barrier effect (ii), the extracts were present only during UV irradiation. In this case, protection was attributed to the UV-absorbing capacity of the bioactives in the surrounding medium, rather than to metabolic activity, since there was no time for ingestion or metabolization. Absorption through the worm’s surface is considered negligible, as the *C. elegans* cuticle acts as a natural barrier preventing compound penetration [33]. Finally, to assess the physiological effect (iii), nematodes were exposed to carob extracts for 24 h, followed by medium removal and washing before UV exposure, ensuring that photoprotection effect was solely due to metabolically mediated mechanisms derived from the prior ingestion of the extracts.

For each condition, approximately 200 synchronized adult nematodes were collected from NGM agar plates by washing with K-medium [34] per well, ensuring no *E. coli* was present during the treatments. The worms were then suspended in the different carob samples previously diluted at 1% and 5% in liquid K-medium, depending on the experimental groups. Control group (without carob extracts) was included in each assay.

UV exposure was performed using a 6 W UVGL-55 hand-held UV lamp (Analytik Jena US LLC, Upland, CA, USA) emitting at 254 nm [20,35,36]. The lamp was positioned at a fixed distance of 15 cm above the samples, delivering an irradiance of 7.45 W/m^2^ for 20 min. Prior to each experimental series, the lamp output and emission spectrum were checked and calibrated according to the manufacturer’s instructions to ensure consistent irradiance and correct wavelength (peak at 254 nm, narrow-band). After irradiation, the number of live and dead nematodes was determined by visual inspection under a stereo dissecting microscope (Motic SMZ-161, Motic China Group CO., Xiamen, China). Nematodes were considered dead if they failed to respond to mechanical stimulation with a metal wire. Optimal stressor conditions were determined in preliminary assays to ensure high mortality in control. Each analysis was conducted in quadruplicate (*n* = 4). The results were expressed as the percentage of dead worms relative to the total number of individuals in each well (Equation (4)).

(4) $$Lethality\;rate\left(\%\right)=\frac{number\;of\;dead\;worms}{number\;of\;total\;worms}\times 100$$

### 2.5. Statistical Analysis

The assumptions of normality and homoscedasticity were verified using the Shapiro–Wilk and Levene tests, respectively. After confirming these assumptions, a one-way ANOVA was applied to the in vitro results, including total phenolic content (TPC), antioxidant activity, sun protection factor (SPF), and erythema transmission, as well as to the in vivo experiments among carob fractions. Tukey’s Honestly Significant Difference test was used for post hoc comparisons. This test is considered conservative and appropriate when comparing multiple groups means (more than four), as it effectively controls the Type I error rate [37]. To assess the association between variables, Spearman’s rank correlation was used. All statistical analyses were conducted using Statgraphics Plus (v. 19.1.2; Stat-Technologies Point Technologies, Inc., Warrenton, VA, USA), with significance set at *p* < 0.05.

## 3. Results and Discussion

### 3.1. Total Phenolic Content

Polyphenols are naturally occurring plant compounds characterized by multiple phenolic groups that confer potent health-promoting and photoprotective properties. Therefore, their quantification is essential to understand the mechanisms underlying photoprotection [38]. Carob is known to contain various polyphenol, including phenolic acids and flavonoids, which contribute to its antioxidant and photoprotective effects [18]. Therefore, assessing total polyphenol content in carob extracts would provide key insights into their potential efficacy as natural photoprotective agents.

**Figure 1:**
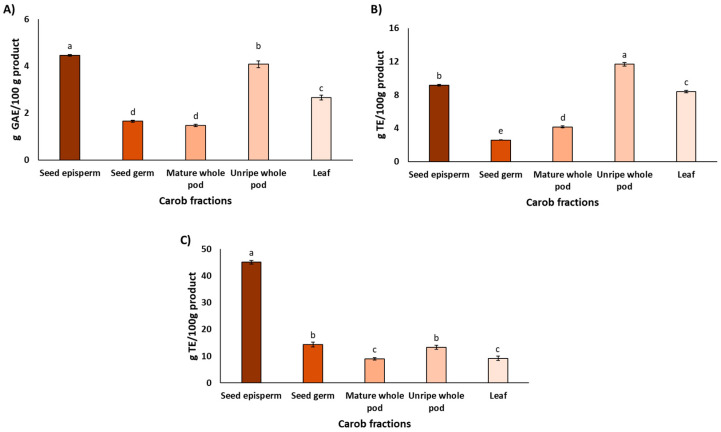
The total phenolic content (**A**) and antioxidant properties assessed by the DPPH assay (**B**) and ORAC assay (**C**) in the different carob fractions. Data are expressed as mean ± SD, *n* = 3. Lowercase letters indicate significant differences among carob fractions (*p* < 0.05).

As shown in Figure 1A, the carob fraction with the highest total polyphenol content was the seed episperm (4.8 ± 0.3 g GAE/100 g product). Relative to this fraction, the unripe pod contained 85%, the leaf 55%, the mature pod 34%, and the seed germ 31%. Statistically significant differences were observed among all fractions (*p* < 0.05), except between the germ and the mature pod. Direct comparison between studies remains challenging due to the strong influence of genetic background, environmental and agronomic conditions, developmental stage at harvest, and methodological differences in extraction and quantification [39].

Nevertheless, analyses of TPC across fractions, when performed within the same study, tend to reveal more consistent patterns. For instance, Rico et al. [40] reported ca. 50% higher polyphenol concentrations in the seed episperm compared with the mature pod. Similarly, Custódio et al. [41] observed approximately 3-fold higher polyphenol contents in carob leaves compared with carob pod. Taking together, these findings support a general trend of higher phenolic accumulation in protective tissues such as seed coats and leaves compared with storage tissues such as mature pods [42], which is consistent with the results of the present study. Complementing these quantitative observations, a recent comprehensive review of the phytochemical composition of carob fractions revealed a remarkable diversity of compounds, with over 50 distinct phytochemicals identified [18]. Gallic acid was consistently reported as the most abundant phenolic acid across all fractions. Among flavonoids, kaempferol was predominant in the pulp, quercetin in both the episperm and germ of the seeds, and myricetin in the leaves. Regarding tannins, digalloyl-glucose and trigalloyl-glucose were notably present in the pulp and both seed fractions. This chemical diversity suggests a broad spectrum of potential bioactive functions.

### 3.2. In Vitro Antioxidant Activity

Measuring antioxidant activity in carob fractions is relevant not only for nutritional and pharmacological purposes but also for understanding their photoprotective functions.

As shown in Figure 1B, the unripe pod and seed episperm exhibited the highest antioxidant activity in the DPPH assay, with the leaf also showing notable values. This distribution aligns with the physiological roles of each tissue, particularly the leaf, which is directly exposed to solar radiation and thus requires strong photoprotective mechanisms to mitigate ROS formation [18,42]. Other tissues, although less exposed, may still accumulate bioactive compounds that contribute to the overall resilience of the plant. It is noteworthy that the seed episperm exhibited elevated antioxidant activity, which is presumably attributable to bioactive compounds that safeguard seed viability during dormancy [43].

Regarding antioxidant activity measured by the ORAC assay, Figure 1C shows that the carob fraction with the highest capacity was the seed episperm (45.1 ± 0.7 g TE/100 g product), consistent with the results obtained using the DPPH assay. However, the relative antioxidant values for the remaining fractions were notably lower in ORAC compared to DPPH. Specifically, ORAC values were 32% for the seed germ, 29% for the unripe pod, and 20% for both the leaf and the mature pod.

As mentioned above, the antioxidant activity of carob fractions, as assessed by the DPPH and ORAC assays, revealed notable differences among extracts. While both the unripe pod and seed episperm exhibited high activity in the DPPH assay, only the seed episperm remained the most potent fraction in ORAC. These discrepancies were confirmed by Spearman’s rank correlation (ρ = 0.1971, *p* = 0.4608), which reflects the distinct chemical mechanisms and solvent environments underlying each method. DPPH operates via single electron transfer (SET) in a lipophilic medium, favoring fast-reacting, electron-donating compounds such as certain flavonoids, whereas ORAC measures hydrogen atom transfer (HAT) capacity against peroxyl radicals in an aqueous buffered system [44,45]. The high ORAC value of the seed episperm likely reflects its richness in condensed tannins and stable polyphenols capable of sustained radical neutralization [46], whereas the unripe pod may contain more reactive flavonoids that perform well in DPPH [47]. Despite these differences, Spearman’s rank correlations showed a strong association between TPC and DPPH (ρ = 0.7742, *p* = 0.0038) and a moderate–strong association between TPC and ORAC (ρ = 0.6464, *p* = 0.0156), indicating that polyphenol content is a major determinant of antioxidant capacity in carob fractions and supporting the role of carob as a valuable source of phenolic compounds contributing to radical scavenging activity through complementary mechanisms.

### 3.3. In Vitro Photoprotective Analysis

#### 3.3.1. Solar Protection Factor

In vitro evaluation of photoprotective properties is a crucial step prior to incorporating plant extracts into topical formulations [48,49]. These assays allow for the relation of intrinsic features such as antioxidant potential, which is essential for predicting the efficacy of the extract under physiological conditions. As demonstrated by Caballero-Gallardo et al. [13], such in vitro assessments provide a rational basis for characterizing active ingredients with proven photoprotective effects, thereby enhancing the functional value and safety of carob extracts.

The SPF values of carob extracts at 1% and 5% are shown in Table 1. Seed episperm and unripe pod reached the highest values (10 and 7.7 at 1%, and 17 and 16 at 5%, respectively), followed by seed germ, leaf, and mature pod. These values are consistent with those of Ayad et al. [16], who reported SPF values ranging from 8.62 to 22.37 in carob extracts at 1 mg/mL, depending on the extraction method. Moreover, the SPF levels achieved by carob fractions are comparable to those of *Tagetes lucida* and *Cymbopogon flexuosus* essential oils (14.7 and 13.4, respectively) [13], indicating that carob by-products reach values considered functionally relevant for botanical extracts.

On the other hand, SPF correlated positively with TPC (ρ = 0.975, *p* = 0.0048). However, fractions with similar SPF but different TPC (e.g., leaf and seed germ) indicate that photoprotection would not be determined solely by phenolic concentration but also by compositional factors such as the distribution of polyphenols subclasses, tannin polymerization, and the presence of pigments or Maillard reaction products [50,51]. The consistency of these results across different studies suggests that the photoprotective potential of carob is robust, although compositional differences between fractions likely explain the variability observed.

#### 3.3.2. Erythema Transmission

Parallel to SPF evaluation, erythema transmission, indicative of cutaneous redness, was also measured at 1% and 5%. As can be seen, Table 2 corroborated the photoprotective potential of carob fractions. Seed episperm and unripe pod exhibited the lowest values at both concentrations, corresponding to the “extra protection” category at 5% [52]. The remaining fractions were classified as “standard protection,” and none as “low protection,” indicating that even at minimal concentrations, carob extracts consistently provide measurable photoprotection. Notably, erythema transmission values showed a strong correlation with TPC (ρ = 0.969, *p* = 0.0059), reinforcing the consistency of in vitro photoprotective results.

Considering these results, carob extracts represent an opportunity for revalorization, as they may serve not only as food ingredients but also as active compounds in cosmeceutical and pharmaceutical formulations, thereby expanding their functional and economic value.

Overall, the in vitro assays indicated that carob extracts, particularly the seed episperm and unripe pod, are rich in polyphenols and display high antioxidant and photoprotective capacity. These results suggest their potential as functional ingredients; however, since in vitro approaches only reflect chemical reactivity and UV absorption under simplified conditions, their actual biological efficacy must be validated in vivo. For this purpose, *C. elegans* was employed as a model to assess photoprotection under physiological UV stress.

### 3.4. In Vivo Photoprotective Analysis Using C. elegans

As mentioned above, the assessment of photoprotection under physiological UV stress was performed by evaluating three different conditions: (1) complete exposure; (2) the barrier effect; (3) the physiological effect (see Section 2.4.2 for more details).

**Figure 2:**
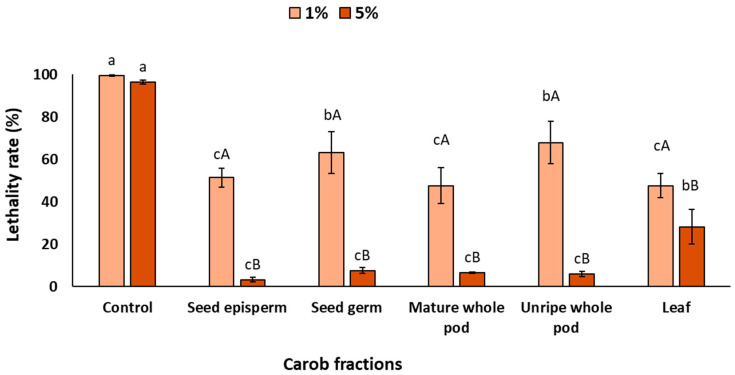
Lethality rate of *C. elegans* exposed to different carob fractions under complete exposure to extracts and UV light. Data are expressed as mean ± SD, *n* = 4. Different lowercase letters indicate significant differences among carob fraction extracts at the same concentration, while uppercase letters indicate differences among extract concentrations within the same carob fraction (*p* < 0.05).

Figure 2 shows the lethality rate of *C. elegans* exposed to UV radiation in the complete exposure condition. As expected, in the absence of carob extracts, lethality reached 98%, confirming the high sensitivity of the nematode to UV-induced oxidative stress. This value corresponds to 0% reduction in lethality, and therefore serves as the baseline against which the protective effect of the extracts was evaluated. After the addition of carob extracts, a significant increase in survival, attributable to their protective effect, was observed (*p* < 0.05). At 1%, seed episperm, mature pod, and leaf provided the strongest protection, reducing lethality by around 50%, whereas the seed germ and unripe pod exhibited comparatively lower protective effects, with reductions closer to 40% (*p* < 0.05). When the concentration increased to 5%, protection was even more remarkable: most fractions reduced lethality by nearly 90%, except for the leaf extract, which displayed a comparatively lower reduction (75%) (*p* < 0.05).

The findings indicate that complete exposure resulted in a significant decrease in mortality under in vivo conditions, which is both promising and encouraging. In this sense, in vitro assays have previously indicated the presence of notable antioxidant and photoprotective properties. However, the employment of in vivo assays has elucidated the efficacy of these bioactive compounds within a complex biological system, where factors such as absorption, metabolism, and cellular responses typically diminish the chemical activity [53]. The fact that carob fractions, particularly the seed episperm, can decrease UV-induced lethality from nearly total mortality (98%) to survival levels above 50% would indicate their extraordinary potential as natural photoprotective agents [54]. According to previous studies that evaluated synthetic compounds or botanical extracts, the photoprotective effect in *C. elegans* would likely be mediated by two complementary mechanisms: (i) direct antioxidant activity that could reduce reactive oxygen species (ROS) generated by UV exposure, and (ii) the possible activation of stress-response signaling pathways that enhance endogenous defense systems [25,26]. Specifically, polyphenols such as resveratrol and flavonoids from tartary buckwheat have been shown to upregulate transcription factors like DAF-16/FOXO and SKN-1/Nrf2, which are central regulators of oxidative stress resistance and longevity in *C. elegans*. These pathways collectively mitigate UV-induced damage by improving cellular redox balance, preserving structural integrity (e.g., collagen), and reducing apoptosis [55,56].

Following the positive results obtained in the trial, we evaluated the underlying protection mechanisms. These included (i) physical protection provided by the surrounding medium, which absorbs or filters part of the UV radiation before it reaches the organism (barrier effect), and (ii) protection derived from biological activity after ingestion and metabolism of the bioactive compounds present in the extracts by the nematode (physiological effect). In this case, the bioactive compounds act systemically, enhancing the organism’s antioxidant defenses and thereby reducing UV-induced lethality.

**Figure 3:**
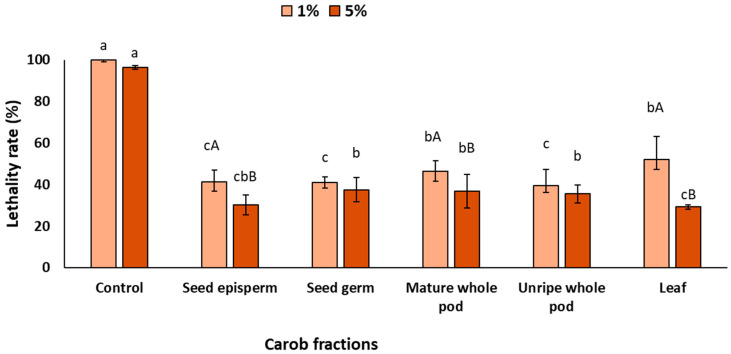
Lethality rate of *C. elegans* exposed to different carob fractions under barrier exposure to extracts and UV light. Data are expressed as mean ± SD, *n* = 4. Different lowercase letters indicate significant differences among carob fraction extracts at the same concentration, while uppercase letters indicate differences among extract concentrations within the same carob fraction (*p* < 0.05).

Figure 3 shows the barrier effects of the different carob extracts after UV exposure. The presence of extracts in the surrounding medium significantly reduced worm lethality at both concentrations, with significant differences among fractions (*p* < 0.05). At 1%, all extracts decreased lethality by at least 45%, with seed germ (58%), seed episperm (57%), and unripe pod (59%) showing the strongest effects, while mature pod (52%) and leaf (45%) exhibited comparatively lower protection. As expected, increasing the concentration to 5% further enhanced UV protection (*p* < 0.05), with seed episperm and leaf reaching around 70%, and seed germ, unripe pod, and mature pod displaying slightly lower but still significant values (ca. 64%).

**Figure 4:**
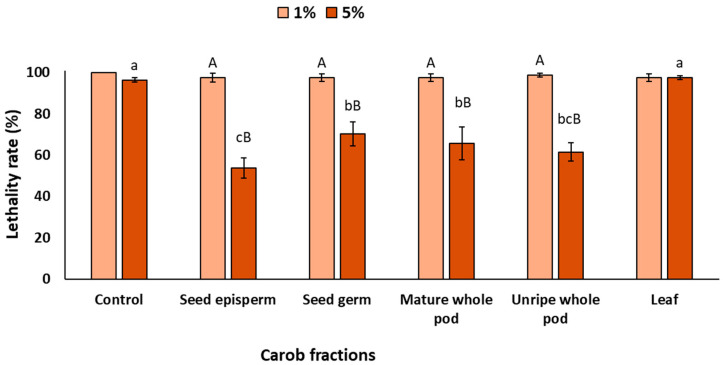
Lethality rate of *C. elegans* exposed to different carob fractions under physiological exposure to extracts and UV light. Data are expressed as mean ± SD, *n* = 4. Different lowercase letters indicate significant differences among carob fraction extracts at the same concentration, while uppercase letters indicate differences among extract concentrations within the same carob fraction (*p* < 0.05).

Once the barrier effect was evaluated, the photoprotective effect resulting from the nematodes’ ingestion of the extracts, also referred to as the physiological effect, was analyzed. As shown in Figure 4, when *C. elegans* ingested carob extracts at 1% prior to light exposure, lethality did not decrease significantly (*p* > 0.05). However, increasing the concentration to 5% led to a significant reduction in lethality, except for the leaf extract, which did not differ from the control (*p* > 0.05). Among the fractions, the seed episperm showed the strongest effect (47% reduction), followed by the unripe pod (39%), mature pod (35%), and seed germ (30%).

Although the magnitude of protection was lower than that observed under complete and barrier exposures, these results are highly relevant, as they would indicate that carob bioactives can be metabolized within the organism and still exert a physiological photoprotective effect, helping to prevent cell death caused by UV radiation. Interestingly, this type of nutritional photoprotection derived from biological activity is consistent with evidence reported for other foods rich in bioactive compounds. For instance, lycopene from tomatoes, β-carotene from carrots, and polyphenols from tea, grapes, or cocoa have all been shown to accumulate in tissues and mitigate UV-induced damage [57,58,59,60]. Such findings support the emerging concept of oral photoprotection or ‘nutricosmetics’, whereby dietary antioxidants contribute to photoprotection after UV exposure.

Taken together, the results suggest that the strong protection observed under complete exposure conditions emerges from the synergistic contribution of both mechanisms: the barrier effect, which prevents part of the UV radiation from reaching the organism, and the physiological effect, whereby ingested compounds are metabolized and exert systemic antioxidant action. Demonstrating that these two processes would act in concert provides a more comprehensive understanding of how carob bioactives confer photoprotection. Importantly, these findings also indicate in a living organism that carob fractions possess remarkable protective capacity against UV-induced damage. However, when extrapolating these results to humans, the study is limited using UVC radiation (254 nm). This wavelength was selected for its high energy, which allowed testing the extracts under the most unfavorable conditions—those generating the strongest oxidative stress—and because it is commonly used to induce oxidative stress in *C. elegans*. Nevertheless, it is not physiologically relevant to humans, as solar radiation mainly consists of UVB (290–320 nm) and UVA (320–400 nm) [26]. This limitation should be considered when interpreting the photoprotective potential of carob extracts under real scenarios.

Furthermore, the use of *C. elegans* as a model system represents a major advantage, as it enables evaluation under realistic physiological conditions that integrate absorption, metabolism, and cellular responses, factors that cannot be captured by in vitro assays alone [21,26]. This also suggests the use of *C. elegans* as a reliable model for testing the bioactivity of specific molecules, particularly those with antioxidant or metabolic functions. Finally, the availability of genetically modified *C. elegans* strains that mimic human metabolic disorders such as obesity and diabetes [61] broadens its applicability, suggesting that this organism could also be employed to assess the bioactivity of carob extracts and other molecules in the context of complex diseases.

Although previous studies have evaluated the photoprotective effects of synthetic compounds and botanical extracts in *C. elegans* [26], to the best of our knowledge, this is the first study to demonstrate the photoprotective activity of carob fractions in *C. elegans*. This biological validation, together with prior research employing *C. elegans* for the functional evaluation of bioactive compounds, reinforces the relevance of the findings and provides robust evidence for the potential application of these extracts in photoprotection. Moreover, given that these fractions, particularly the seed episperm and leaf, are currently considered low-value by-products with limited industrial use [18], our results underscore their potential for revalorization and integral exploitation of a local and underutilized crop. Finally, this approach is consistent with the SDGs, as it promotes the revaluation of underutilized industrial products through the creation of natural and sustainable photoprotective agents.

## 4. Conclusions

This work provides the first comprehensive demonstration of the photoprotective potential of underutilized carob (*Ceratonia siliqua*, L.) fractions, particularly seed episperm and unripe pods. The in vitro analyses revealed high polyphenol content, strong antioxidant activity, and relevant photoprotective properties, including elevated sun protection factors and low erythema transmission values. These findings were further validated in vivo using *Caenorhabditis elegans*, where complete exposure to both extracts and UV radiation significantly reduced lethality rates, from 98% to below 50%, due to the dual contribution of barrier and physiological antioxidant effects.

The consistency between in vitro and in vivo results confirms the biological relevance of carob polyphenols, supports their potential application as natural photoprotective agents, and highlights the opportunity to revalorize low-value carob by-products as sustainable ingredients for cosmetic and nutraceutical applications. This strategy aligns with circular economy principles and the Sustainable Development Goals by promoting innovation, sustainability, and value creation from agricultural residues.

Despite promising data, to support future standardization and reproducibility, further studies should include detailed chemical characterization of each carob fraction, incorporate UV wavelengths relevant to human exposure and include a known antioxidant or UV-protective compound (e.g., resveratrol, quercetin, or ascorbic acid) as a benchmark. Additionally, complementary in vivo endpoints such as reproduction, reactive species production, and other antioxidant biomarkers should be evaluated, as survival data alone cannot confirm photoprotective activity. Finally, the use of *C. elegans* as an in vivo model entails inherent limitations, such as differences in cuticular structure, metabolism, and UV response compared to humans, which restrict direct extrapolation to human photoprotection.

## Figures and Tables

**Table 1:** Sun Protection Factor (SPF) values of carob fractions at concentrations of 1% and 5%. Data are expressed as mean ± SD, *n* = 3.

	Extract Concentration	
Carob Fractions	1%	5%
Seed episperm	10 ± 0.2 ^aB^	17 ± 0.3 ^aA^
Seed germ	7.2 ± 0.1 ^cB^	14 ± 0.3 ^cA^
Mature whole pod	5.7 ± 0.2 ^dB^	9.5 ± 0.2 ^dA^
Unripe whole pod	7.7 ± 0.1 ^bB^	16 ± 0.3 ^bA^
Leaf	7.2 ± 0.1 ^cB^	14 ± 0.1 ^cA^

Different lowercase letters indicate significant differences among carob fraction extracts at the same concentration, while uppercase letters indicate differences among extract concentrations within the same carob fraction (*p* < 0.05). While 1% concentration corresponds to 800 μg lyophilized carob fraction/mL extract, 5% corresponds to 4000 μg lyophilized carob fraction/mL extract.

**Table 2:** Erythema transmission values of carob fractions at concentrations of 1% and 5%. Data are expressed as mean ± SD, *n* = 3.

	Extract Concentration	
Carob Fractions	1%	5%
Seed episperm	5.5 ± 0.4 ^dB^	2.8 ± 0.2 ^dA^
Seed germ	6.8 ± 0.5 ^cB^	3.6 ± 0.5 ^cA^
Mature whole pod	10.0 ± 0.4 ^aB^	5.8 ± 0.5 ^aA^
Unripe whole pod	7.5 ± 0.3 ^cB^	3.9 ± 0.5 ^cA^
Leaf	9.3 ± 0.4 ^bB^	4.9 ± 0.5 ^bA^

Different lowercase letters indicate significant differences among carob fraction extracts at the same concentration, while uppercase letters indicate differences among extract concentrations within the same carob fraction (*p* < 0.05). While 1% concentration corresponds to 800 μg lyophilized carob fraction/mL extract, 5% corresponds to 4000 μg lyophilized carob fraction/mL extract.

## Data Availability

Data are contained within the article.
